# Solution Structures of Anionic–Amphoteric Surfactant Mixtures near the Two-Phase Region at Fixed pH

**DOI:** 10.1021/acs.langmuir.2c00527

**Published:** 2022-06-03

**Authors:** Gunjan Tyagi, William N. Sharratt, Sofia Erikson, Dale Seddon, Eric S. J. Robles, João T. Cabral

**Affiliations:** † Department of Chemical Engineering, Imperial College London, London, SW7 2AZ, UK; ‡ The Procter & Gamble Company, Newcastle Innovation Centre, Newcastle-Upon-Tyne NE12 9TS, UK

## Abstract

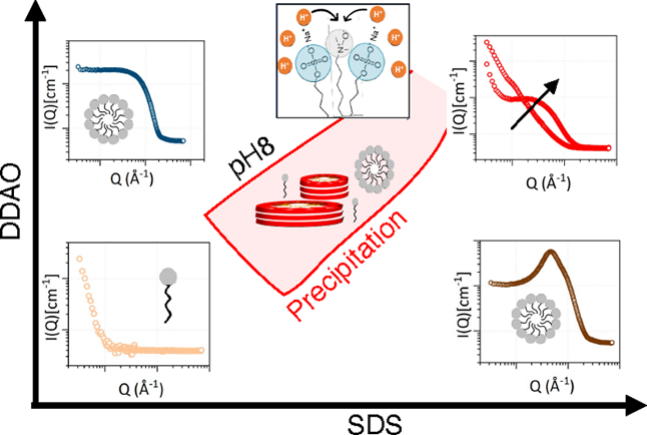

We examine the solution structures in a mixed surfactant system of sodium dodecyl sulfate (SDS) and *N*,*N*-dimethyldodecylamine *N*-oxide (DDAO) in water, on both sides of the two-phase boundary, employing dynamic light scattering, small-angle neutron scattering, and Fourier transform infrared spectroscopy. The precipitate phase boundary was accessed by lowering pH to 8, from its floating pH 9.5 value, and was experimentally approached from the monomeric and micellar regions in three ways: at fixed DDAO or SDS concentrations and at a fixed (70:30) SDS:DDAO molar ratio. We characterize the size, shape, and interactions of micelles, which elongate approaching the boundary, leading to the formation of disk-like aggregates within the biphasic region, coexisting with micelles and monomers. Our data, from both monomeric and micellar solutions, indicate that the two phase structures formed are largely pathway-independent, with dimensions influenced by both pH and mixed surfactant composition. Precipitation occurs at intermediate stoichiometries with a similar SDS:DDAO ratio, whereas asymmetric stoichiometries form a re-entrant transition, returning to the mixed micelle phase. Overall, our findings demonstrate the effect of stoichiometry and solution pH on the synergistic interaction of mixed surfactants and their impact on phase equilibrium and associated micellar and two-phase structures.

## Introduction

Surfactants are key constituents of formulations underpinning a wide-range of industries including personal care,^1^ oil and lubricants,^2^ food,^3^ and agriculture.^4^ Mixtures of surfactants are generally employed to tune solution structure, stability, and interfacial performance.^5,6^

Mixtures of surfactants whose head groups are oppositely charged and electrostatically interact, i.e., anionic and cationic or amphoteric surfactants, can lead to synergistic interactions,^7,8^ able to improve surface activity and thus foaming, wetting, and detergency.^9^ Synergistic interactions are often inferred from a reduction in the critical micelle concentration (CMC), a shift in micelle shape and size (often increasing in size), large viscosity increases, and shifts in the temperature-concentration equilibrium phase boundaries between micelles and other mesoscopic phases.^10^ However, their utility is constrained by a propensity to precipitate, in the presence of oppositely charged ions or surfactants, at specific solution conditions.

The current theoretical description of precipitation in mixed surfactant systems^11^ considers surfactants in three different arrangements: as monomers below the CMC, incorporated in mixed micelles above CMC, and as a precipitate, when the monomeric concentration of both the anionic and cationic monomers exceed their solubility product (*K_sp_*). Surfactant stoichiometry, monomer–micelle equilibria, temperature, and pH are known to impact the precipitation behavior of mixed surfactant solutions. Since precipitation is driven by electrostatic interactions between charged surfactant head groups, it is critical to understand the effect on precipitation of changes to the solution pH. For systems with added inert salt and fully dissociated surfactants, pH appears to play a minor role in determining the precipitation boundary;^12^ however, pH is expected to have a substantial effect on precipitation when the charge of either surfactant depends on it (e.g., for amine oxides and betaines).

Amine oxide surfactants exhibit properties of cationics at low pH values (pH <p*K_a_* ∼ 5) and non-ionics at higher pH values since they are protonated by hydrogen ions in a similar manner to weak acids,^13^ i.e., at a given pH, an equilibrium is established between non-ionic and cationic forms of the surfactant.^14^ Thus, the mixture of an amine oxide surfactant with an anionic surfactant effectively becomes a ternary system containing anionic surfactant, cationic surfactant (protonated), and nonionic surfactant (unprotonated), resulting in synergism in both micelle formation and decreased CMCs. Solution properties of the amine oxide surfactants have been shown to vary with pH and added electrolyte concentration.^15−17^ Since the fraction of charged species of a pH-sensitive surfactant depends on the hydrogen ion concentration, solution pH plays an important role in defining their precipitation behavior. The precipitation of anionic surfactants has previously been investigated using a variety of mono and/or divalent cations^18,19^ and oppositely charged surfactants.^12,18,20^ Additionally, models for predicting precipitation region have also been developed,^11,12,21,22^ combining regular solution theory (monomer–micelle equilibrium) with a solubility product relationship (monomer–precipitate equilibrium) and have been shown to fit well to experimental data. Experimental systems, which form additional self-assembled structures, *e.g.*, coacervates or vesicles, cannot be modeled as effectively and require an additional empirical correction to describe the measured phase boundaries.^12^ Due to intricate mixed surfactant interactions, the presence of complex structures in the precipitate region constrains the scope of theoretical modeling. Thus, it becomes critical to experimentally determine and characterize the solution structures, in both the single- and two-phase regions, across the phase boundary, over a practically relevant pH range.

**Figure 1:**
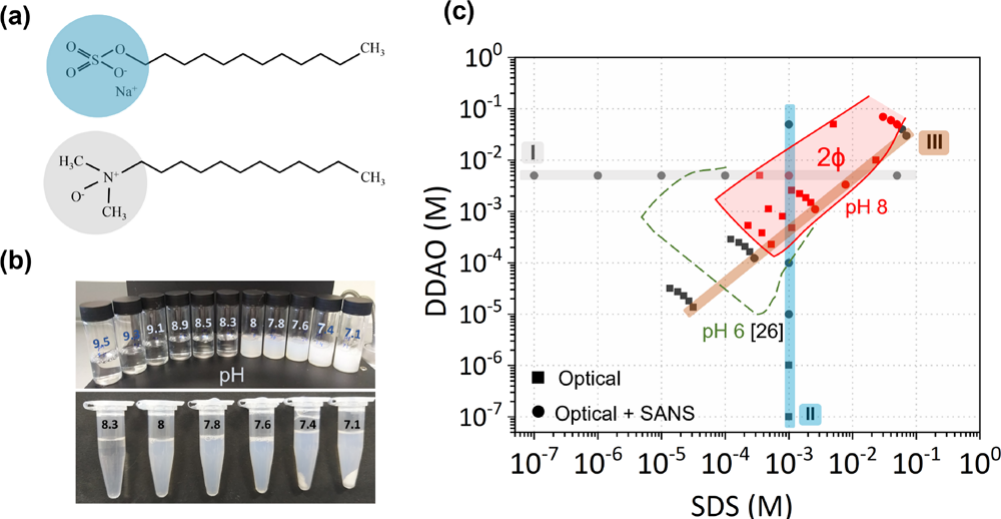
(a) Structure of sodium dodecyl sulfate (SDS) and *N*,*N*-dimethyl dodecylamine *N*-oxide (DDAO). (b) Clear to turbid transition of equimolar SDS–DDAO solutions (100 mM) upon decreasing pH (top row). Upon centrifugation, a precipitate appears at pH 7.4–7.1 (bottom row, reproduced with permission from ref (25). Wiley Online Library, 2017). (c) Composition space investigated for SDS–DDAO mixtures at fixed pH 8: series (I) fixed DDAO (5 mM) and varying SDS concentration; (II) fixed SDS (1 mM) and varying DDAO concentration, and (III) fixed SDS–DDAO molar ratio of 70:30. Red shaded area encloses the two-phase, optically turbid, region (2ϕ). Literature data at pH 6 taken from ref (26) shown by the dashed line.

In this paper, we select mixtures of sodium dodecyl sulfate (SDS) and dodecyldimethylamine oxide (DDAO) in aqueous solution as a model anionic/amphoteric surfactant mixture, whose chemical structures are shown in Figure 1a. The role of pH is particularly relevant for such systems, as the protonation state of the amphotheric surfactant is non-trivially influenced by the presence of the anionic surfactant. As such, solution pH varies with concentration and stoichiometry,^23^ in turn affecting the solution structures and the delicate stability of the mixture. In most practical applications, this pH “self-regulation” is impacted by chemical environments with distinct pH that perturb the solution structures and equilibrium, potentially causing precipitation and thus performance loss. For SDS/DDAO mixtures, the pH sensitivity of DDAO largely determines its synergistic interaction with SDS and the mixed-system phase behavior. Further, pH values outside 6–8 cause chemical instability in mixtures of alkyl sulfates, due to expedited breakage of the sulfate ester bond.^24,25^

We have previously demonstrated that protonation of DDAO can be altered by SDS, impacting mixed micelle formation and adsorption at the air–water interface at “floating” pH conditions.^23^ In the absence of added acids/buffers, mixing of SDS and DDAO results in an increase in solution pH from neutral to alkaline (>7). pH reduction by acid addition has a clear effect on the optical appearance and alters the nano- and microscale structures of surfactants in solution (illustrated in Figure 1b). Precipitation phase boundaries have previously been experimentally determined and theoretically modeled for SDS–DDAO mixtures in the pH range 4–6.^26^ Here, we seek to examine the solution structures formed by imposing a (fixed) pH below the spontaneous pH state (8–9.5) of the system. We select a representative value of pH 8, in order to induce solution instability across a considerable concentration range, enabling us to resolve the solution structures on both sides of the phase boundary. We combine three complementary techniques: dynamic light scattering (DLS) to estimate the hydrodynamic size of micelles and precipitated structures, small-angle neutron scattering (SANS) for the precise determination of their size, shape, and interactions, and Fourier transform infrared (FTIR) spectroscopy for the understanding of their molecular packing.

## Materials and Methods

### Materials

Sodium dodecyl sulfate (NaC_12_H_2_SO_4_, SDS, >99.0% purity), *N*,*N*-dimethyldodecylamine *N*-oxide (C_14_H_31_NO, DDAO), hydrochloric acid (HCl), and deuterium oxide (D_2_O) were purchased from Sigma-Aldrich and used as received. For SANS, DDAO in powder was used, while for turbidity measurements, a 30 wt % aqueous (H_2_O) solution was employed. Solutions were prepared by diluting the surfactants in deionized water (for DLS and FTIR) and D_2_O (SANS). Water used was obtained from a PURELAB Chorus 1 ultrapure water system from ELGA LabWater, delivering water purity of 18.2 MΩ cm. The surface tension of pure water was measured to be 71.5–72.0 mN/m at 25 °C.

### Methods

#### pH Measurements

The pH of the solutions was monitored using a Hanna Edge pH and conductivity meter and adjusted with dilute HCl to pH 8. In practice, in order to prepare a solution with prescribed surfactant concentration and pH 8, initial surfactant stock solutions of higher (∼20–50% higher) concentration were made and their pH was adjusted to 8, by dropwise addition of 0.1 M and then 0.01 M HCl solutions; water was then added to obtained the desired surfactant concentration, and the pH value confirmed. Details of solution compositions and HCl addition are provided in the Supplementary Information, Table S1.

#### Dynamic Light Scattering

Correlograms and size distributions were obtained from measurements on a time-resolved optical fiber dynamic light scattering instrument (VASCO KIN, Cordouan Technology, Pessac, France). Approximately 5 mL of each solution was measured directly in glass vials with the instrument *in situ* head, which operates with a detector angle of ∼170°. For each sample, the instrument laser power was tuned for each sample to maximize the measured coherence (β value) of the correlogram prior to measuring for ∼30 s per sample. Intensity correlograms were analyzed via a sparse Bayesian learning, which provided the lowest residual values across the correlogram, with the instrument’s NANO KIN software. Reported size distributions are intensity-weighted, and values discussed are the mean values of the peaks.

#### Fourier Transform Infrared Spectroscopy

Infrared spectra were recorded using an INVENIO-S FTIR spectrometer with a DTGS detector and Platinum ATR accessory. For each spectrum, 64 single beam scans were averaged with 4 cm^–1^ resolution in the range of 4000 to 600 cm^–1^. The clean, dry diamond crystal was consistently used for background correction. Results were examined in the absorbance unit using OPUS 8.5 software. Spectral subtraction of water and standard baseline correction were performed on all the spectra and analyzed with no further data processing.

#### Small Angle Neutron Scattering (SANS)

SANS measurements were performed on the D22 diffractometer (ILL, Grenoble, France) in quartz cells (1 mm banjo, Hellma) with incident neutron wavelength λ = 6 Å and Δλ/λ = 10%. The instrument operates with two detector banks with sample-to-detector distances of *d*_1_ = 17.6 and 5.6 m and *d*_2_ = 1.3 m, which cover a *Q*-range of 0.0032–0.70 Å^–1^. Samples were measured at *T* = 25 °C, maintained by a water both connected to the sample rack. Data were reduced and corrected for solvent, cell, and background scattering according to standard procedures in GRASP (Lockdown V9.31). The reduced data, scaled in absolute units, were analyzed in SASView (v5.0.4). Scattering profiles from mixed SDS–DDAO micellar solutions were fitted with a core–shell ellipsoidal form factor and a Hayter–Penfold RMSA structure factor. Turbid samples could not be well described by this model. Instead, a cylindrical form factor, with a radius larger than its length (i.e., forming a disk) could describe all datasets. A structure factor RMSA was required to model the weakly turbid samples (SD5, 9), while the form factor alone sufficed to describe the remainder (SD12, 13). While the RMSA is strictly valid for spherical particles, it can approximate interactions of non-spherical objects at comparatively large interparticle distances;^27^ since the maximum total surfactant concentration investigated was 100 mM (<3%), we have assumed its validity. Within SASView, we have selected the RMSA’s effective radius to be estimated from the “average curvature” of spheroids and “excluded volume” for cylinders. Both models were implemented by fixing the scattering length densities (SLD) of the pure components SDS (core and shell, respectively −0.489 and 1.5 × 10^–6^ Å^–2^) and DDAO (core and shell, respectively −0.091 and 0.603 × 10^–6^ Å^–2^) and their weighted average for intermediate concentrations; we fitted volume fraction, micellar charge, and micellar/disk dimensions as free parameters, ensuring self-consistency with solution concentration, and dependence between charge and concentration or aggregation number. Error bars in dimensions and charge (particularly large within the turbid region) are obtained from the range of fitting parameters compatible with the scattering data.

## Results and Discussion

**Figure 2:**
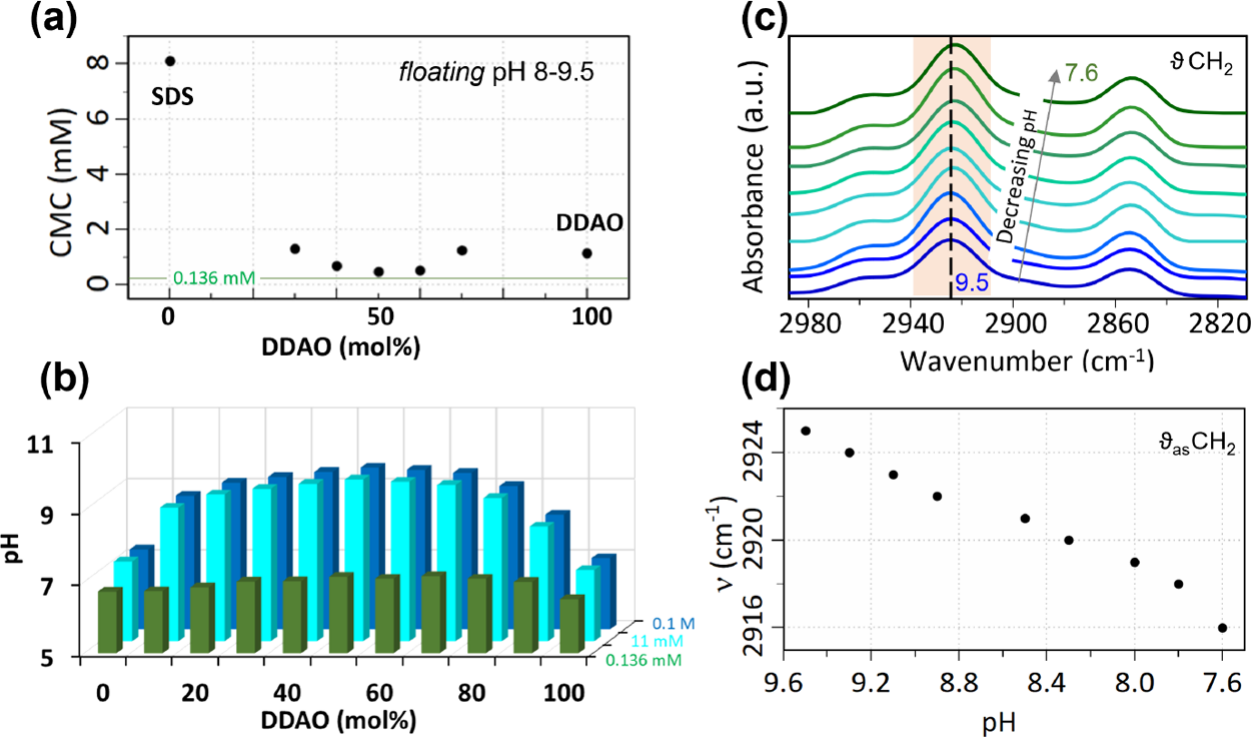
(a) CMC for SDS–DDAO mixtures measured at floating by pendant drop tensiometry.^23^ The horizontal green line corresponds to 0.136 mM, below CMC at all ratios. (b) Solution pH of SDS–DDAO mixtures at 25 °C for three total surfactant concentrations above and below CMC. (c) ATR-FTIR spectra of equimolar SDS–DDAO solutions at 25 °C and decreasing pH values from 9.5 (floating) to 7.6, by HCl addition. (d) Frequency of antisymmetric C–H stretching peak of hydrocarbon tails as a function of pH.

The precipitation phase boundary for SDS–DDAO solutions at fixed pH 8 was determined by investigating the phase behavior across a wide range of mixed surfactant concentrations. The corresponding solution structures were mapped, with DLS and SANS, on both sides of the phase boundary. As illustrated in Figure 1c, we approached the phase boundary from three directions; a fixed DDAO concentration (I), a fixed SDS concentration (II), and a fixed molar ratio (III). The CMC and pH values of mixed SDS–DDAO solutions, as a function of surfactant molar ratio, are reported in Figure 2a,b, respectively. The CMC decreases for all ratios, relative to the pure surfactant solutions, with a minimum value at a 1:1 stoichiometry (data previously reported in ref (23)). The pH of the mixed solutions increases from neutral values of the pure surfactants to alkaline values (>7), indicating protonation of the DDAO and a reduction of free H^+^ ions, and reaches a maximum at 9.5 for equimolar surfactant ratios. Lowering the pH by HCl addition causes precipitation and turbidity, starting at near-equimolar surfactant ratios. CMC measurements at pH 8 cannot therefore be carried out across all surfactant ratios. However, we have found that, wherever measurable, CMC values at lower, fixed pH did not vary considerably from those reported here.^23^ As an example, the CMC of equimolar surfactant solutions at pH 7.5 was found to be 0.43 mM, only slightly below the 0.47 mM measured at floating pH (pH 9.5); this solution eventually precipitates at 7.2–7.3 pH. We therefore examined the effects of pH on the solution structure using FTIR (and subsequently DLS and SANS). FTIR spectra characterizing the changes in molecular packing for the equimolar mixture of SDS and DDAO upon decreasing pH from 9.5 to 7.6, by HCl addition, are shown in Figure 2c. The C–H stretching frequency of surfactant tails significantly decreases upon decreasing pH, which confirms the effect of pH on solution structures by driving surfactant tails to transition from a predominately gauche to a trans geometry (Figure 2d).

To assess the phase behavior and location of the phase boundary, the pH of mixed SDS–DDAO solutions was adjusted to 8 from their floating pH value, and phase change was initially determined visually. DLS and FTIR were used to estimate the size and molecular arrangement of surfactant structures in solution, and SANS was used to determine their precise shape and size within the single and the two-phase region.

### Approaching Phase Boundary from Mixed Surfactant Series I

**Figure 3:**
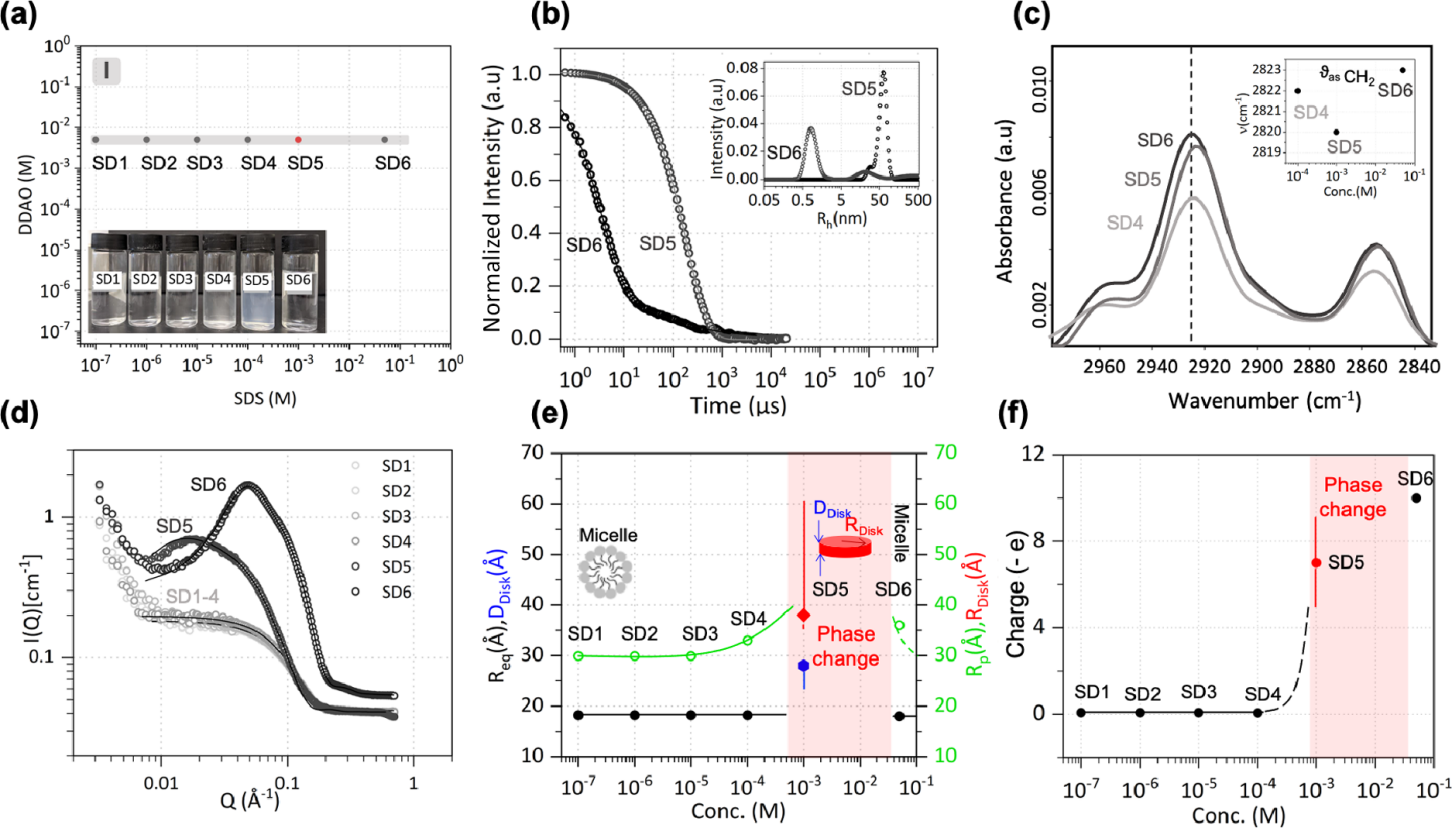
(a) SDS–DDAO series I, measured at pH 8 and 25 °C. Inset image shows optical appearance of the solutions. (b) DLS data for solutions SD5 and SD6; intensity-weighted distribution of hydrodynamic radii *R_h_* shown in inset. (c) ATR-FTIR spectra of mixed solutions SD4–6, depicting shift observed for the C–H stretching region of surfactant tails (inset). (d) Radially averaged SANS data and model fit for solutions SD1–6. (e) Fitted radii of the ellipsoidal micelle model (SD 1–4 and 6) and disk model (SD5). (f) Fitted total net charge of micelles and disks. Red shaded areas denote the 2ϕ region.

Solutions marked as SD1–6 comprising a fixed DDAO concentration (5 mM) and varying SDS concentration (0.0001–50 mM) were adjusted to pH 8 with the addition of HCl, and their physical appearance was monitored. Compositions and an optical image are shown in Figure 3a. All solutions bar SD5 appeared entirely optically transparent. SD5 underwent a visible color change to a faintly, light blue turbid solution, which is typically associated with the formation of a small number of nanoscale objects (>20 nm).

#### Dynamic Light Scattering

DLS was used to investigate the hydrodynamic size of the surfactant structures responsible for the transition between faintly turbid SD5 and optically transparent SD6 solutions (Figure 3b). The second-order autocorrelation function of scattered light is shown for the two samples as a normalized intensity (in arbitrary units). The turbid sample (SD5) has a single, broad decay at relatively long decay times while the optically transparent solution appears with a fast decay and a small tail corresponding to a very small population of near micrometer-sized objects. The intensity-weighted size distributions determined by data fitting with a sparse Bayesian learning (SBL) algorithm are shown as an inset in Figure 2b. The main decay of SD5 is centered around a mean hydrodynamic radius *R_h_* of 58 nm, while the prominent SD6 decay is centered around 9 nm with two smaller populations (<2% by number) with larger decays, 29 nm and 1.3 μm, likely resulting from the equilibrium aggregation of micelles and agglomerate formation.

#### Fourier Transform Infrared Spectroscopy

Vibrational responses from mixed surfactant solutions were measured by FTIR spectroscopy to examine the molecular arrangement of solution structures for samples SD4, SD5, and SD6. Figure 3c summarizes the results obtained from FTIR measurements, which all exhibit strong absorption bands arising from methylene groups in the tail (3000–2800 cm^–1^) of the surfactants. Several vibrational modes are sensitive to distinct aspects of molecular conformation and interaction of surfactant molecules, and thus changes in peak frequency and shape can characterize structural transitions. The methylene stretching frequencies 2920 and 2855 cm^–1^ correspond to antisymmetric and symmetric stretching of C–H bonds, respectively, and can be used to qualitatively monitor both conformational order and acyl chain packing.^28^ The sensitivity to conformational order (*trans*–*gauche* isomerization in the chains) is well known and is used to describe monomer-to-micelle transformations^29^ or coagel to micelle changes.^30^ Kakitani *et al*.^31^ examined the sphere-to-rod transition of SDS and DDAO mixed micelles, and a frequency shift of the same order (2–4 cm^–1^) was observed as found here across the stability boundary.

The position of the SD5 C–H peak was observed at lower wavenumber as compared to SD4 and SD6. Figure 3c inset shows the variation of antisymmetric wavenumbers as a function of total mixed surfactant concentration. The results exhibit a shift from higher frequency (high energy), characteristic of gauche conformation and associated with chain disorder, to lower frequency (low energy), which is characteristic of an ordered trans conformation. The decrease in frequency (of 3 cm^–1^) can be attributed to the compaction of tail methylene groups, which for SD5 transition into a trans geometry. This tighter packing could be attributed to an increase in aggregation number and corresponding increase of size as compared to SD4 and SD6, which exhibit C–H stretching frequencies similar to those of micellar structures.

#### Small-Angle Neutron Scattering

SANS measurements were used to examine the structure of mixed surfactant solutions in both one-phase and two-phase regions. The total scattering profiles of mixed surfactant series I are shown in Figure 3d. For this series, all compositions are expected to be above the CMC of the solution, given the DDAO concentration is fixed at a value above the CMC of DDAO in solution.^23^ The SANS profiles of solutions with SDS concentrations ≪ DDAO concentrations (SD1–4) appear characteristic of non-ionic, dilute micelles. However, as the concentration of SDS increases and becomes comparable to DDAO (∼mM), as seen in samples SD5–6, the intensity of the mid-*Q* region peak increases and is characteristic of charged micelle structure factor. SANS profiles for SD 1–4 and SD6 showed good fits to a prolate ellipsoid model. For SD6, the structure factor was modeled with the Hayter–Penfold rescaled mean spherical approximation (RMSA), indicating the presence of intermicellar interactions between charged species. The SANS profile of SD5, which is observed to be two-phase, is more accurately described by a disk-like shape, which we fit with a cylinder model with comparable length, denoted *D*_disk_, and radius, denoted *R*_disk_. We hypothesize that the changes in molecular structure and head group interactions, characterized by FTIR, cause the structural changes in micelle nanostructure. All data show an upturn in the low-*Q* region, indicating the presence of aggregate structures in addition to micelles. Additionally, after appropriate background subtraction, SD5 and SD6 exhibit a power law of *Q*^–4^, indicating the formation of a well-defined sharp interface. We interpret it as the formation of objects of large dimensions (Figure S1). Through the combination of optical appearance, DLS and SANS data suggest SD6 comprises a narrow-size distribution of micelles, although the appearance of a low-*Q* power law for the coherent SANS data may indicate its proximity to the phase boundary. Figure 3e shows the fitted structural parameters from both ellipsoidal and cylindrical (discoidal) models. The error bars correspond to the uncertainties estimated by the maximum range of dimensions compatible with the data. As the phase boundary is approached, the size and aspect ratio of micelles increases. The equatorial radius of the micelle *R*_eq_ remains constant while the polar radius *R*_p_ increases. As the SDS concentration increases to 1 mM (SD5), the system becomes optically turbid and two-phase, and the constituent micelles appear to transition from ellipsoidal to discoidal. The fitted disk radius, which can provide acceptable fits over the range 35–60 nm, corroborates well the long decay time and conformational change for surfactant tails observed by DLS and FTIR measurements, respectively. As the SDS concentration is increased further, the solution appears single-phase (SD6) and is made up of charged mixed SDS–DDAO micelles, which are prolate ellipsoidal in shape.

The DDAO-rich micelles exhibit a modest size increase as they approach the phase boundary. The findings are consistent with a study of micellar aggregation numbers for a mixture of SDS and dodecyltrimethylammonium chloride (DTAC), a cationic–anionic mixture.^32^ The p*K_a_* of DDAO monomer has been reported to be ∼5,^33^ but the effective p*K_a_* can be as high as 1 pH unit higher for pure DDAO micelles^34−36^ and about 2.5 units higher for mixed micelles. Electrostatic coupling of DDAO and SDS in mixed micelles shifts the DDAO protonation equilibrium toward the protonated form and consequently releases OH^–^ ions and increases solution pH.^37,33^ For such micelles, the additive effect of lowering the solution pH (by adding additional H^+^ ions) further increases the concentration of the cationic form of DDAO within the micelles. From SD1 to SD4 with low concentration of SDS (0.0001–0.01 mM), only a slight increase in size is observed. By increasing the SDS concentration further by a factor of 10 with a molar ratio of 5:1 DDAO:SDS at SD5, it is expected that the high-concentration SDS^–^ leads to strong electrostatic interaction between DDAO^+^ and SDS^–^ and results in the formation of larger, disk-like micelles, which can aggregate and induce phase separation in the solution. A similar effect on the precipitate structure has been previously reported and appears to be governed by the molar ratios of anionic and cationic surfactants in the mixture.^38^

Increasing the solution concentration of SDS to 50 mM (SD6) results in the formation of SDS-rich mixed micelles as inferred from the large structure factor contribution to the SANS scattering profile, which is characteristic of interaction between negatively charged micelles. The fitted charge for samples SD1–6 illustrates this and is shown in Figure 3f. This charge, within the Hayter–Penfold RMSA model, corresponds to the effective repulsion between charged spheroids with a given separation. Owing to the relative molar ratio of SDS:DDAO, the DDAO monomer–micelle equilibrium is shifted and we expect that DDAO^+^ is predominantly contained in mixed micelles. The free monomeric concentration of DDAO is insufficient to precipitate, and so we traverse the phase boundary and observe an optically clear solution comprising SDS-rich micelles.

### Approaching Phase Boundary from Mixed Surfactant Series II

**Figure 4:**
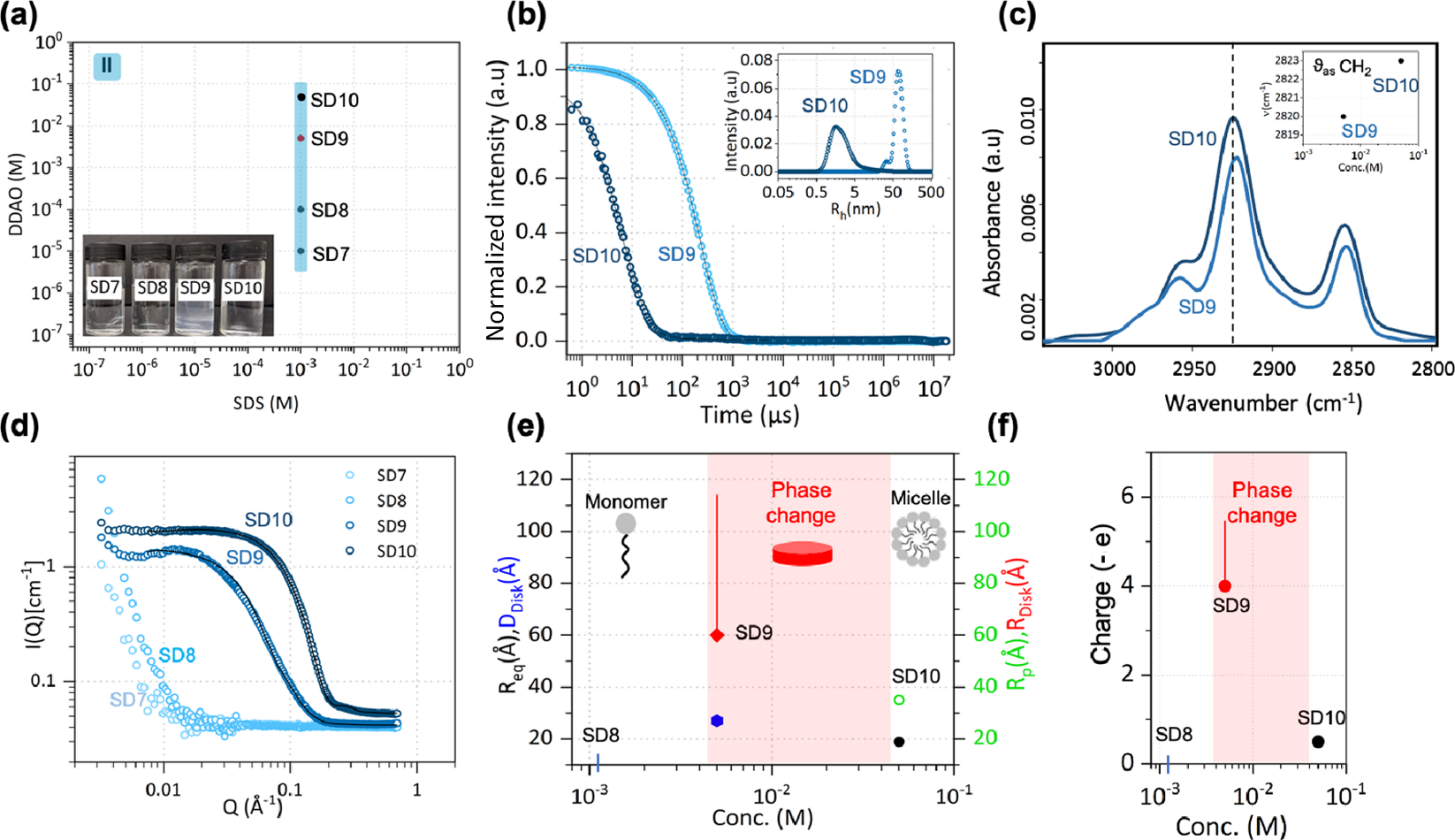
(a) SDS–DDAO series II, starting from the monomer region, at pH 8. Inset shows the optical appearance of the solutions. (b) DLS data for solutions SD9 and SD10; intensity-weighted distribution of *R_h_* shown in inset. (c) ATR-FTIR spectra of SD9–10 depicting the shift in the C–H stretching region of surfactant tails (inset). (d) SANS profiles of solutions SD7–10 and model fits to ellipsoidal micelles (SD10) and disks (SD9), while SD7 and SD8 are monomeric. (e) Corresponding dimensions and (f) total net charge. Red shaded area denotes the 2ϕ region.

SD7–10 solutions containing a fixed concentration of SDS (1 mM) and a variable concentration of DDAO (0.01–50 mM) were adjusted to pH 8 with HCl, and their physical appearance was monitored. As illustrated in Figure 4a, SD9 exhibited a visible phase change signified by the appearance of turbidity.

#### Dynamic Light Scattering

DLS was used to investigate the hydrodynamic size of the surfactant structures responsible for the transition between faintly turbid SD9 and optically transparent SD10 solutions (Figure 4b). Both samples appear to have single decay in the autocorrelation function but with distinct differences in decay times. The optically transparent sample, SD10, has a relatively short decay time while the faintly turbid SD9 has a much longer one. The intensity-weighted size distributions are shown as an inset in Figure 4b. SD10 is centered around a mean *R_h_* of 2.9 nm, while SBL fitting yields two populations contributing to the SD9 decay: a small shoulder at 32 nm and a main peak at 73 nm. While the DLS instrumental configuration cannot decouple translation and rotation from hydrodynamic motion measured, the SBL fitting indicates two key length scales, which may correspond to discrete populations of aggregate structures.

#### Fourier Transform Infrared Spectroscopy

Spectroscopic evidence for the C–H stretching regions of the spectra for mixed surfactant solution indicates a constrained environment for the surfactant tails, accompanying the structural transition (Figure 4c) and likely increase in micelle size and aggregation number. This is the same observation as when the two-phase region is approached at fixed DDAO concentration and increasing SDS concentration (series I). The inset shows that the frequency of the antisymmetric CH_2_ band exhibits a decrease within the two-phase region. The changes can be interpreted as indicating a decrease in the gauche/trans conformer ratio, *i.e.*, a partial ordering of the surfactant tails, within the ordered structures, which accompany a change in micelle shape, aggregation, and phase separation.

#### Small-Angle Neutron Scattering

Figure 4d shows the SANS profiles from mixtures SD7–10 and model fits, where appropriate. At a mixed total surfactant concentration of 0.01 mM (SD7) or 0.1 mM (SD8), which is lower than the CMC of either surfactant, the scattering shows a largely flat *Q*-independent signal ∼0.04 cm^–1^. This value is indicative of the solvent D_2_O and implies the absence of micelles in solution. The SANS profiles obtained when DDAO concentration was increased to 5 mM (SD9) and 50 mM (SD10) are indicative of the presence of micelles in solution. For both SD9 and SD10, an ellipsoidal form factor and Hayter MSA structure factor modeled the data well, and the fitted structure parameters are shown in Figure 4e. However, similar to SD5, within the two-phase region, a disk-like shape gave better agreement with the data and provides a more satisfactory model for SD9. Given that both SD5 and SD9 arrive at the same concentration via two distinct routes, if they were an equilibrium phase, they would be expected to have the same solution structures. The slight difference in their scattering profiles, on the other hand, could be attributed to the evolution of these out-of-equilibrium structures over time during the experiment. Specifically, the low-*Q* region (*Q* < 0.01 Å^–1^) exhibits an increase in intensity, indicating aggregate growth, which is likely to vary with time.

In series II, the phase boundary is approached from SDS and DDAO concentration well below their respective CMC values and we observed the distinct lack of micelles present for both SD7 and SD8. It is known that monomeric SDS has no significant effect on protonation of DDAO and therefore, given the solution pH = 8 > p*K_a_* ∼ 5, DDAO remains in its nonionic form and no precipitation is observed. Further increasing the concentration of DDAO to 5 mM, the surfactant concentrations reach the same molar ratio as SD5. The same features are observed for SD5 as SD9; turbidity of the solution, shift in C–H stretching frequency, long decay time in DLS correlograms (and corresponding large *R_h_*) alongside the respective SANS profiles suggest the formation of aggregated disks. This highlights the role of the increased degree of protonation of DDAO^+^ and the resulting stronger electrostatic interactions with SDS, which lead to the solution transition into the two-phase region, independent of the pathway approached.

Thus, while the aggregate size and structure may be temporally evolving, the presence of a two-phase region appears to be an equilibrium feature. After increasing the DDAO concentration further, to 50 mM, the relatively low concentration of SDS was insignificant to undergo precipitation. The majority of SDS molecules are incorporated into mixed micelles and, similar to SD6, a clear solution is formed. In this case, a lack of apparent structure factor peak in the SANS profile is indicative that, owing to the high DDAO molar ratio in the mixed system, micelles remain largely in the unprotonated form. The evolution of charge, extracted from the fitted structure factor, illustrates this and is shown in Figure 4f.

### Approaching Phase Boundary from Mixed Surfactant Series III

**Figure 5:**
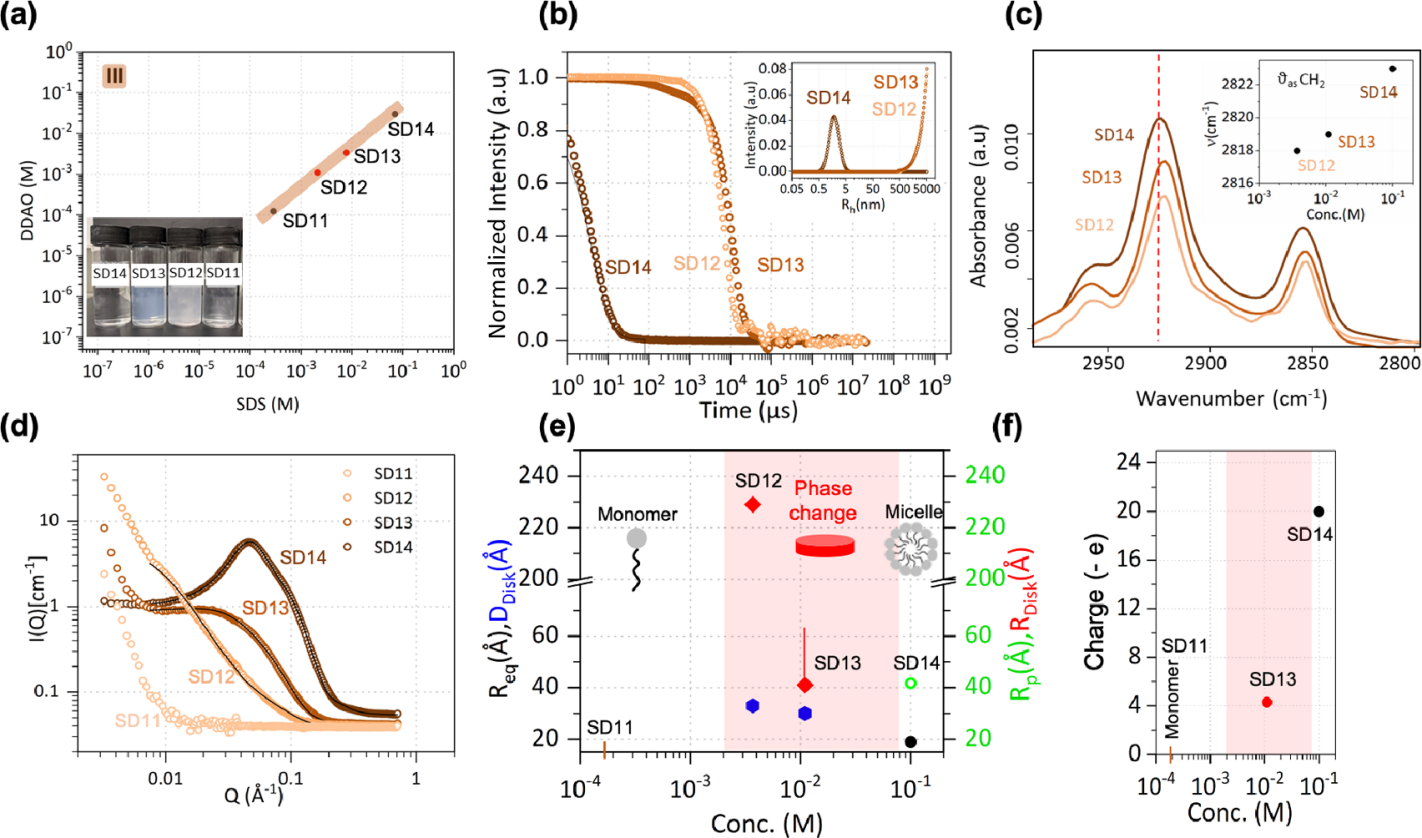
(a) SDS–DDAO (molar ratio 70:30) series III at pH 8; inset shows the optical appearance of the solutions. (b) DLS data for solutions SD12–14, with *R_h_* distribution shown in the inset. (c) ATR-FTIR spectra for SD12–14, depicting the shift observed in the C–H stretching region of the surfactant tails (inset). (d) SANS data and model fit. (e) Radii obtained from fitting SD14 data to ellipsoidal micelles model and SD12 and SD13 to a disk model. (f) Fitted charge of surfactant structures. Red shaded area denotes the 2ϕ region.

Solutions SD11–SD14 contain mixed surfactant with a molar ratio of 70:30 SDS:DDAO with increasing total surfactant concentration. As illustrated in Figure 5a, SD12 and SD13 exhibit a visible change in optical appearance upon decreasing the pH to 8. SD12 appears faintly opaque and white, while the turbidity with SD13 appears faintly blue.

#### Dynamic Light Scattering

Figure 5b shows the autocorrelation functions from samples SD12–SD14 and extracted intensity size distributions shown as an inset. Sample SD14 appears entirely homogeneous and appears with a single, fast decay, while the optically turbid SD12 and SD13 samples both appear turbid and with more complex, long decaying correlograms. SD14 appears with a size distribution centered around *R_h_* = 1.2 nm. The optically turbid samples both appear with size distributions at the upper end of the measurable size range by the VASCO KIN DLS instrument (∼10 μm). However, the correlograms show noticeable differences at shorter timescales. SD13 has a small contribution from a faster decay. By constraining the fitting algorithm to shorter timescales (<10^4^ μs), a small population with *R_h_* = 55 nm can be discerned (not shown in the inset). This suggests a coexistence between a small population of nanoscale objects with the larger micron-scale structure, while the SD12 sample, which optically appears turbid (white) rather than a faint bluish color, has a correlogram dominated by the presence of micron-scale precipitates.

#### Fourier Transform Infrared Spectroscopy

The mixtures were found to be consistent with the previous two-phase samples in series I and II but with a more pronounced downward shift of the C–H stretching for SD12 (Figure 5c). This appears to coincide with the largest aggregate structure observed with DLS. The highest wavenumber of antisymmetric C–H stretching is observed for SD14 at 2823 cm^–1^, which is typical of surfactant tails arranged in ellipsoidal micelles in a gauche conformation.^39^ At lower total surfactant concentrations (SD13 = 11 mM) and (SD12 = 3.7 mM), the arrangement of surfactant tails shifts to a more compact trans conformation, as indicated by a 4 and 5 cm^–1^ decrease in antisymmetric stretching for SD13 and SD12, respectively. A 5 cm^–1^ decrease in vibrational frequency for antisymmetric tail stretching indicates that geometric constraints have shifted the orientation of surfactant molecules within the micelles. These changes should also be reflected in the surfactant head group’s vibrations. S–O antisymmetric vibrations were examined in detail and revealed a change in the shape and position of the S–O doublet for samples SD12 and SD13 (Figure S2), suggesting the presence of comparatively larger structures with straight surfactants tails in a *trans* geometry and close interaction of surfactant head groups.

#### Small-Angle Neutron Scattering

SANS spectra of SD11 to SD14 are shown in Figure 5d. It is evident that there are significant differences in the scattering profiles as a function of concentration at this fixed molar ratio. Below the CMC (SD11), as estimated from our previous study,^23^ the profile is typical of scattering from solvent alone and implies the absence of micelles. As concentration is increased, the phase boundary is crossed and the scattering profile from SD12 exhibits a significant low-*Q* upturn with no mid-*Q* micellar peak. A micellar signal is observed for SD13 and SD14 with a significant, sharp upturn observed at low-*Q* for SD13. For surfactant concentrations in the single-phase region and above the CMC (SD14), an ellipsoidal model adequately describes the SANS intensity. For samples that show turbidity, SD12 and SD13, a cylindrical form factor with dimensions characteristic of disks fits the data better. Fitted micelle dimensions and micelle charge, extracted from the structure factor, are shown in Figure 5e,f, respectively. The lack of the mid-*Q* correlation peak in SD12 is indicative of the lack of intermicellar interactions, likely owing to the low overall surfactant concentration. Additionally, the associative interaction between DDAO^+^ and SDS^–^ and subsequent neutralization of the charge lower the average area occupied by the surfactant head groups. A closer head group packing is evident from the perturbation of S–O stretching frequency, making the formation of disks more energetically favorable. The large radii (60–220 Å) extracted for SD12 and low-*Q* power law are also consistent with DLS estimates of large aggregate structures in the micron range. The approach to and across the phase boundary with a fixed molar ratio of surfactant is also alike series II.

The evolution of structures and precipitate formation as they approach the two-phase region as well as the precipitate dissolution into clear solution after crossing the phase boundary emphasizes the importance of surfactant stoichiometry and solution pH, particularly in formulations containing pH-sensitive surfactants.

### Composition Analysis of Two-Phase Region

To further understand the composition of phase-separated solutions, we centrifuged them and observed the separation of the supernatant and precipitate for SD12 and SD13 and not in the case of SD5 and SD9. This suggests that the aggregate size varies within the two-phase region and with proximity to the phase boundary. The sizes of structures formed by SD5 and SD9 (30–60 nm), estimated from SANS and DLS are 3 times smaller than those of SD12 (35–220 nm) and therefore do not sediment when centrifuged. Two factors play a role in defining the two-phase region: (i) at a fixed pH below the native, “floating” pH increases the H^+^ ion concentration in solution and leads to a greater fraction of protonated DDAO present in the mixed micellar structures, and (ii) at intermediate stoichiometries, with similar molar ratios of SDS and DDAO, electrostatic coupling of surfactants within the micelles neutralizes the micelle charge and reduces intermicellar repulsion. This results in a shape change in constituent micelles as well as the formation of large aggregates, which lead to visible turbidity. Variation in the size and shape of precipitated structures has also been seen before for another anionic–cationic surfactant mixture, SDS and cetylpyridinium chloride (CPCL), as the molar ratio of SDS to CPCl was increased.^38^

**Figure 6:**
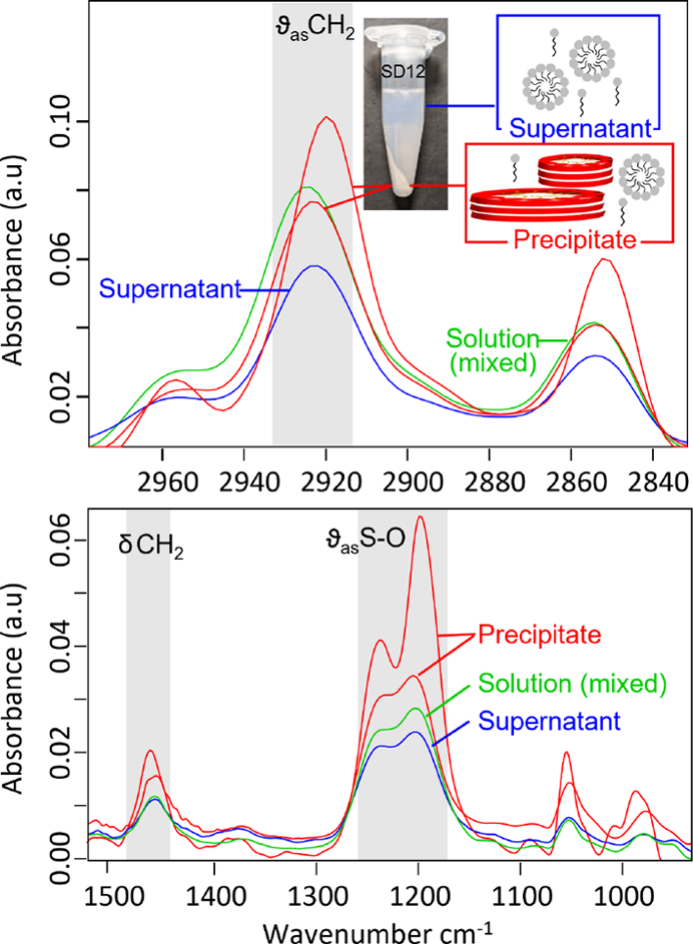
Representative ATR-FTIR spectra of a 2ϕ surfactant solution, SD12, before (mixed solution) and after centrifugation (supernatant and precipitate) at pH 8. Top panel shows the C–H stretching region of hydrocarbon surfactant tail; bottom shows C–H bending region of the tail and the antisymmetric S–O stretching region of the sulfate head group. Schematics depict disks and aggregates in the precipitate along with micelles and monomers, whereas the supernatant consists of micelles and monomers.

FTIR spectra of the solution prior to centrifugation, the resulting supernatant, and the precipitate after centrifugation are shown in Figure 6. Analysis of the precipitate reveals the presence of highly ordered tails with a downward shift of 7 cm^–1^ for CH_2_ stretching frequency as well as a high wavenumber shift and an increase in frequency (1470 cm^–1^) for CH_2_ bending vibrations. The frequency of the CH_2_ bending or “scissoring” band is dependent upon the methylene chain packing and conformation. Fully disordered liquid-like chains exhibit a considerably broadened and relatively lower intensity scissoring band between 1468 and 1466 cm^–1^, as observed for micellar structure of surfactants,^29^ while high frequencies (1470–1472 cm^–1^) suggest ordering of the methylene chains approaching that of a crystalline geometry. This can be pictured as an extended all-*trans* conformation, which contains a few *gauche* defects that are mostly near the chain ends.^40,41^ Further changes in the asymmetric mode of the S–O stretching band, which depends upon the environment and interaction of head groups, are indicative of changes in surfactant–surfactant interactions and useful in detailing the structural alterations.

Two overlapping peaks in the region of 1200–1240 cm^–1^ describe the directionality of the S–O interaction and correspond to the antisymmetric and symmetric S–O stretching modes. They are extremely sensitive to head group interactions parallel and perpendicular to the head group surface. The precipitate spectrum of SD12 shows an asymmetric S–O band near 1263 cm^–1^ accompanied by another low intensity major band near 1205 cm^–1^. The changes in the symmetric mode also show perturbations, presumably because of the electrostatic coupling between surfactants and the proximity of the SDS and DDAO head groups. The major changes associated with vibrational modes of surfactant head groups are consistent with the frequency shifts observed for both stretching and bending modes of CH_2_, showing increased methylene chain straightening. The vibrational mode perturbations provide insight into the molecular structural arrangement; the precipitate in the two-phase region has vibrational signatures typical of a more crystalline and ordered material. The precipitate spectra shown in Figure 6 suggest that the material may be heterogeneous in nature. Additionally, the spectra of solutions and supernatant exhibit similarity in their vibrational responses and imply that micelles and large aggregate structures coexist in the two-phase region.

## Conclusions

We have experimentally investigated the solution structure and phase equilibrium of a model anionic–amphoteric mixed surfactant system, namely, SDS:DDAO in water, focusing on the evolution across the single-phase (micellar and monomeric) and two-phase region, or a precipitate loop. The SDS:DDAO system is synergistic, for instance, exhibiting a large decrease in surface tension and CMC at intermediate molar ratios, with the minimum at approximately 1:1 SDS:DDAO. The presence of SDS leads to the electrostatic coupling and enhanced protonation of DDAO in mixed micelles, accompanied by an increase in solution pH with the maximum at equimolar ratios of SDS and DDAO, owing to the release of OH^–^ ions back into solution. Decreasing pH, by acid addition, below this native “floating” pH value leads to the opening and widening of a biphasic region at intermediate SDS:DDAO ratios. From a practical standpoint, this is problematic as it causes aggregation and surfactant precipitation. This behavior is rationalized in terms of the increased fraction of protonated DDAO in the monomeric form and within mixed micelles. In turn, higher DDAO^+^ concentration increases electrostatic interactions with SDS^–^. At intermediate stoichiometries, this eventually results in solution demixing and the formation of a two-phase region, which widens toward lower overall surfactant concentrations.

In order to elucidate the solution structures in concentration ranges approaching and within the biphasic region, we have employed a combination of DLS, SANS, and FTIR. Specifically, three routes were considered, from the micellar and monomeric phase, intersecting the phase boundary. At fixed DDAO concentration, approximately 5 times above its neat CMC, and increasing SDS concentration (series I), micelles elongate as they approach the two-phase region and transition into elongated disks (∼5 nm) up to larger aggregates (∼50 nm) within the two-phase region. A decreased CH_2_ frequency of surfactant tails suggests a gauche/trans conformational change and ordering. We interpret this change in molecular packing to be responsible for the nanoscale structural change from prolate ellipsoids to disk, as estimated by SANS. Within the two-phase region, disks form and aggregate into large structures that coexist with micelles and monomers.

The two phase region was also accessed via composition pathways originating from the monomer phase, specifically at fixed SDS concentration, increasing DDAO concentration (series II), and a fixed molar ratio of 70:30 SDS:DDAO (series III). While the CMC at a 70:30 molar ratio is found to be 1.1 mM (at pH∼9), the solutions demixed at a concentration of 1.4 mM at fixed pH 8, therefore near the (un-adjusted) CMC conditions from the monomeric phase. Importantly, the presence of disks in the two-phase region when approached from either direction indicates that the two-phase structures are pathway-independent. Variation in the size of the solution structures in the two-phase region is influenced by both pH and composition of both surfactants and proximity to the phase boundary. Solutions containing larger aggregates (from ∼20 nm up to micron range) precipitated upon centrifugation. Changes in the surfactant tail and head group vibrational spectra are indicative of highly ordered structures, akin to crystals. The results suggest that monomers, micelles, disks, and crystals coexist in the two-phase zone. The two-phase region forms a re-entrant transition and returns to a single phase comprising mixed micelles. This occurs when the SDS:DDAO molar ratios are asymmetric and the resulting micelles are either SDS-rich and charged or DDAO-rich and uncharged. Our findings provide insight into the composition space and structure of the species responsible for the precipitation loop in mixed surfactant systems under the effect of pH and elucidate the nature of undesirable behavior in synergistic anionic/amphoteric surfactant mixtures, relevant to their practical use.

## Supporting Information Available

Background subtracted SANS intensity I-B against the scattering variable *Q* for samples SD4-SD6; FTIR spectra showing variation in head group frequencies (1200–1260 cm^–1^) for samples SD12, SD13, and SD14; and table providing solution details, including the amount of HCl added to fix pH 8 for all samples (PDF)
