# Supplementary material for: Solution Structures of Anionic–Amphoteric Surfactant Mixtures near the Two-Phase Region at Fixed pH
Source: Langmuir. 2022 Jun 3;38(23):7198–207. doi: 10.1021/acs.langmuir.2c00527 (PMC9202344; doi:10.1021/acs.langmuir.2c00527)
Supplement: Supplementary file 1 — la2c00527_si_001.pdf [file la2c00527_si_001.pdf]

Supporting Information

Solution structures of anionic-amphoteric surfactant mixtures near the two-phase region at fixed pH

Gunjan Tyagi[a,*], William N. Sharratt[a], Sofia Erikson[a], Dale Seddon[a], Eric S. J. Robles[b], João T. Cabral[a*]

[a]Department of Chemical Engineering, Imperial College London, SW7 2AZ London, United Kingdom
[b]The Procter & Gamble Company, Newcastle Innovation Centre, NE12 9TS Newcastle-Upon-Tyne, United Kingdom

*Email: g.tyagi@imperial.ac.uk (Gunjan Tyagi), j.cabral@imperial.ac.uk (João T. Cabral)

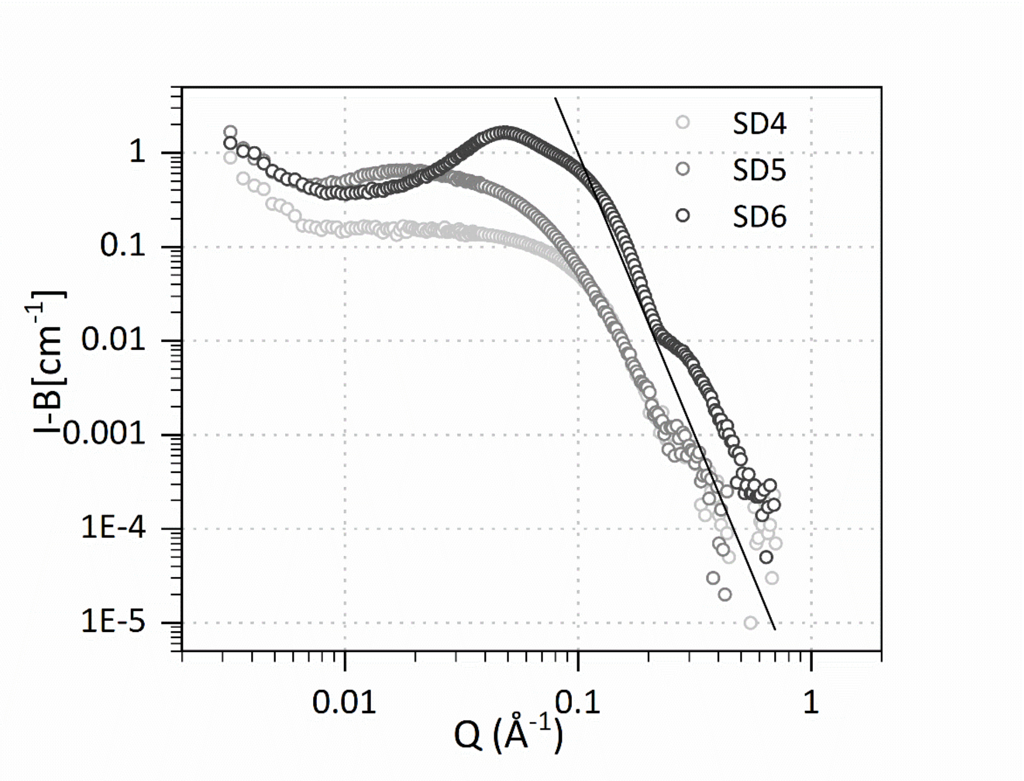

Fig. S1: Background subtracted SANS intensity I-B plotted against the scattering variable Q for samples SD4-SD6. The solid line represents the fit to power law for the samples SD5 and SD6. The samples follow a power law of q-4, suggesting the formation defined interfaces which is interpreted as formation of large objects.

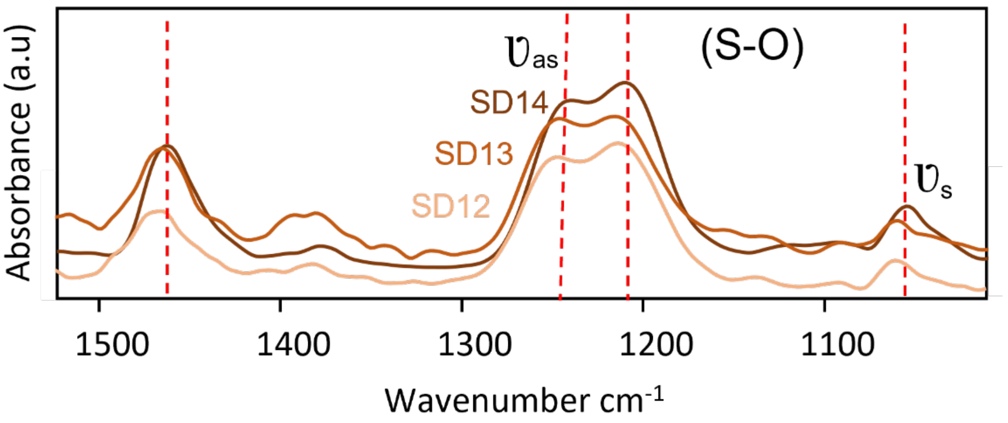

Fig. S2: FTIR spectral region from 1200-1260 cm^{-1} depicting the sulphate headgroup peaks where changes are primarily observed in the transition dipole moment vector for S-O anti-symmetric stretching mode for SD12 and SD13 solutions as compared to micellar features observed for SD14 solution.

Table S1: Details of solution composition and HCl addition.

				HCl (μl)	
Sample	SDS conc. (mM)	DDAO conc. (mM)	Total volume (ml)	0.01M	0.1M
SD1	5.00	0.0001	20	20	-
SD2	5.00	0.001	20	20	-
SD3	5.00	0.01	20	30	-
SD4	5.00	0.1	20	50	-
SD5	5.00	1	20	-	115
SD6	5.00	50	20	-	500
SD7	1.00	0.01	20	10	-
SD8	1.00	0.1	20	20	-
SD9	1.00	5	20	-	155
SD10	1.00	50	20	-	300
SD11	0.28	0.12	20	30	-
SD12	2.6	1.11	20	-	100
SD13	7.7	3.3	20	-	300
SD14	70.00	30.00	20	-	1500

All the solutions are prepared by using stock solution of SDS and DDAO of varying concentrations (1M to 1mM), mixed in required amount to reach the desired total concentration in 20ml of sample volume.
